# Acute Primary Gastric Tuberculosis

**Published:** 1913-01

**Authors:** 


ACUTE PRIMARY GASTRIC TUBERCULOSIS. Dr. Norman P. Geis, *L. I. Med. Jour.,* Sept. 1912, presents the following case history: A man 37 years of age in January, 1909, presented himself with a tumor of the mediastinum. His symptoms were difficulty of breathing, high pulse rate with palpitation of the heart, dizziness and loss of strength and weight. Finally after consultation this tumor was thought to be a gumma. It disappeared under the iodides in six months. He remained well till August, 1911. On August 1, 1911, he arrived in Mexico and remained there till December 1, 1911, coming to me on the 7th of this month. After being in Mexico one month he began to show signs of stomach distress after eating, with gas and a full feeling. This kept up till October when vomiting was added. This vomiting took place one-half hour after eating. This continued during the month so that he restricted his diet to liquids and semi-solids. During November he vomited after each meal if he took more than eight ounces of food at a time. Pain began this month to be continuous when the stomach was empty. Food would relieve the pain for about an hour. His diet is malted milk and once a day a poached egg. The pain is in the region of the pyloric end of the stomach and the duodenum. He never vomited blood. Constipated. Loss 33 pounds in four months. A doctor in Mexico said he had a dirty stomach and the X-ray showed a spot in the liver to the left of the median line. In Mexico all meat is eaten the day it is killed. There is a distinct rigidity of the rectus on the right upper end with an indefinite mass under same. Some tenderness was also found. Test meal gave HCl 5 and total acidity 13 with some blood and only half of the meal returned. No blood in stools. Methylene blue test of urine positive on two tests. My diagnosis was cancer of the gastro-duodenal junction or an ulcer of the same with stenosis in either event. Operation—The first 5 inches of the duodenum was enlarged to the size of a banana and hard to the touch. Four-fifths of the stomach was involved in a hard mass that was ¾-inch in thickness. Owing to the man's poor condition, a posterior no loop gastro-jeiunostomy was considered the only operation of choice. It was difficult to find a clear place in the stomach for my union. It was necessary to bring the summit of the greater curvature of the stomach down to get this free space. A section of the stomach was removed for examination.

He made an easy recovery and left the hospital in two weeks. One month after operation he had gained 6 pounds and was free from pain and vomiting. Pathological report—Tuberculosis of the mucous membrane of the stomach. No syphilis. Cancer not found but was not excluded for full section of stomach was not turned over to pathologist.

